# Stress and Inflammation in Coronary Artery Disease: A Review Psychoneuroendocrineimmunology-Based

**DOI:** 10.3389/fimmu.2018.02031

**Published:** 2018-09-06

**Authors:** Massimo Fioranelli, Anna G. Bottaccioli, Francesco Bottaccioli, Maria Bianchi, Miriam Rovesti, Maria G. Roccia

**Affiliations:** ^1^Department of Nuclear Physics, Sub-Nuclear and Radiation, Guglielmo Marconi University, Rome, Italy; ^2^Società Italiana di Psiconeuroendocrinoimmunologia, Rome, Italy; ^3^Department of Internal Medicine, Sapienza University, Rome, Italy; ^4^Department of Clinical Medicine, University of l'Aquila, L'Aquila, Italy; ^5^Department of Neurosciences “Rita Levi Montalcini”, University of Turin, Rome, Italy; ^6^Department of Dermatology, University of Parma, Parma, Italy

**Keywords:** coronary disease, stress, inflammation, immune system, cytokines, atherosclerosis, psychoneuroendocrineimmunology

## Abstract

Recent findings have deeply changed the current view of coronary heart disease, going beyond the simplistic model of atherosclerosis as a passive process involving cholesterol build-up in the subintimal space of the arteries until their final occlusion and/or thrombosis and instead focusing on the key roles of inflammation and the immune system in plaque formation and destabilization. Chronic inflammation is a typical hallmark of cardiac disease, worsening outcomes irrespective of serum cholesterol levels. Low-grade chronic inflammation correlates with higher incidence of several non-cardiac diseases, including depression, and chronic depression is now listed among the most important cardiovascular risk factors for poor prognosis among patients with myocardial infarction. In this review, we include recent evidence describing the immune and endocrine properties of the heart and their critical roles in acute ischaemic damage and in post-infarct myocardial remodeling. The importance of the central and autonomic regulation of cardiac functions, namely, the neuro-cardiac axis, is extensively explained, highlighting the roles of acute and chronic stress, circadian rhythms, emotions and the social environment in triggering acute cardiac events and worsening heart function and metabolism in chronic cardiovascular diseases. We have also included specific sections related to stress-induced myocardial ischaemia measurements and stress cardiomyopathy. The complex network of reciprocal interconnections between the heart and the main biological systems we have presented in this paper provides a new vision of cardiovascular science based on psychoneuroendocrineimmunology.

## Introduction

Until a few years ago, atherosclerosis was considered a “lipid storage disease,” and it was expected that aggressive pharmacological treatment of hypercholesterolemia couldvirtually eliminate coronary artery pathologies. However, despite an intensive campaign against classical risk factors, cardiovascular disease remains the first cause of death worldwide, with an increasing prevalence in developing countries.

The notion that coronary artery disease can be considered an inflammatory disturbance emerged in the late 1990s (1, 2). Inflammation plays a pivotal role throughout all atherogenesis steps: from foam cell accumulation to fatty streak organization and fibrous plaque formation, until acute plaque fissuring, rupture, and thrombosis.

New insight into atherosclerosis as a complex multifactorial condition highlights the importance of an excessive inflammatory response in the pathogenesis of the fibro-proliferative reaction in the subintimal arterial space and subsequent thrombus formation following various forms of injurious stimuli, leading to an acute coronary event (3, 4).

Common cardiovascular risk factors, such as a high saturated fat diet, smoking, hypertension, hyperglycaemia or insulin resistance, tend to produce chronic inflammation that leads to endothelial activation through impaired nitric oxide (NO) production and loss of vasodilatory and antithrombotic properties of the coronary endothelium (3, 4).

One of the main interests in current cardiovascular research is the identification of inflammatory markers and cellular molecular pathways underlying atherosclerotic diseases in order to develop strategies for prevention and therapy.

## New vision of coronary heart disease: beyond the concept of cholesterol

Although Rudolf Virchow had already recognized the inflammatory nature of atherosclerotic plaques in the nineteenth century (5), coronary artery disease was traditionally considered a cholesterol storage disorder characterized by the progressive accumulation of cholesterol and thrombotic debris in the artery wall. A considerable number of published clinical and epidemiologic studies linked high cholesterol levels to increased risk of cardiovascular events. In particular, a metanalysis of clinical trials investigating the effects of inhibitors of cholesterol synthesis (i.e., statins) established a reduced risk of coronary heart disease with reductions in the LDL cholesterol concentration (6).

In a large clinical trial it was observed that serum high-sensitivity C-reactive protein (hs-CRP), the principle marker of underlying systemic inflammation, was a significant predictor of cardiovascular risk, even in a subgroup of women with low LDL cholesterol (7).

Epidemiological studies (8) and prospective clinical trials (9, 10) have also shown an increased risk of cardiovascular events in patients with high levels of CRP irrespective of cardiovascular risk assessment and lipid profiles, highlighting a key role for inflammation in atherosclerotic disease.

A higher CRP level also seems to correlate with a recurrent risk of myocardial infarction, incidence of sudden death (11) and peripheral arterial disease (12) in patients with acute coronary syndrome (13, 14). Similar results were obtained with other inflammatory markers such as interleukin-6 (IL-6) and serum amyloid A (SAA) (7, 12).

Based on this clinical evidence, The Working Group for Disease Control and Prevention and the American Heart Association suggested the introduction of hs-CRP measurement as a screening practice in all patients for the routine assessment of cardiovascular risk in order to identify asymptomatic patients without any known cardiovascular disease who may be at higher risk than that estimated by traditional risk factors for acute cardiac events (15).

Specifically, assessment of CRP may be helpful in those patients at intermediate risk (i.e., 10–20% calculated risk of coronary heart disease (CHD) over 10 years) to guide further clinical evaluations and start a therapeutic programme.

**Figure 1:**
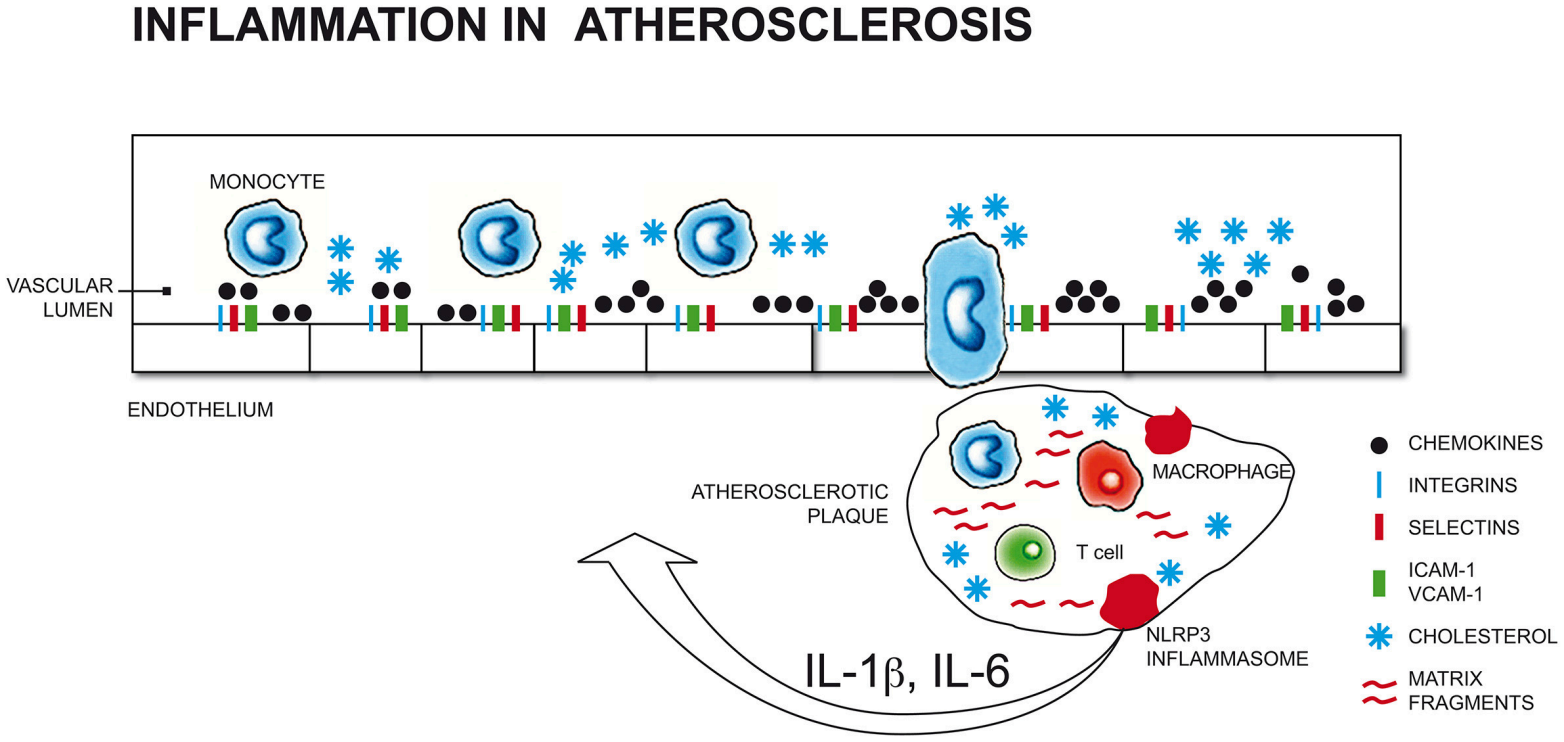
Pathological conditions that include common cardiovascular risk factors (such as hypertension, hyperlipidaemia, hyperglycaemia, smoking) and stress-related conditions (i.e. depression, anxiety) can elicit immune responses that promote the secretion of leukocyte adhesion molecules and chemotactic factors, inducing monocyte adhesion to endothelial cells and transmigration into the subintimal space. Initial atherosclerotic lesions begin with the differentiation of monocytes into macrophages that engulf cholesterol-rich oxidized lipoproteins to become foam cells that organize into fatty streaks. The perpetuation of pro-inflammatory and oxidizing atherosclerotic stimuli results in the recruitment of further macrophages, mast cells, and activated T and B cells that increase vascular lesions, which, in turn, releasing cytokines (i.e., IL-1β, TNF-α), increases monocytes migration into the subintimal space (17, 18, 19, 20). ICAM-1, Intercellular Adhesion Molecule 1; VCAM-1, Vascular cell adhesion protein 1; NLRP3, nucleotide-binding domain and leucine-rich repeat containing (NLR) family member pyrin domain-containing protein 3; IL-1β, Interleukin 1 beta; IL-6, Interleukin 6.

All stages of the atherosclerotic process might be viewed as an inflammatory response to vascular injury (16) (Figure 1). Pathological conditions that include common cardiovascular risk factors, such as hypertension, hyperlipidaemia, hyperglycaemia and smoking, can elicit immune responses that promote the secretion of leukocyte adhesion molecules and chemotactic factors, inducing monocyte adhesion to endothelial cells and transmigration into the subintimal space.

Initial atherosclerotic lesions begin with the differentiation of monocytes into macrophages that engulf cholesterol-rich oxidized low density lipoproteins (LDL-ox) to become foam cells that organize into fatty streaks. The perpetuation of pro-inflammatory and oxidizing atherosclerotic stimuli results in the recruitment of further macrophages, mast cells, and activated T and B cells that increase vascular lesions.

The fibrous atherosclerotic plaque cap maintains its stability due to interstitial collagen. Cytokines and inflammatory immune cells interfere with the integrity of the collagen matrix, impairing the synthesis of new collagen fibers and stimulating the reabsorption of existing ones through the production and activation of specific enzymes (metalloproteinases); this process makes the cap weaker and prone to rupture. The broken cup exposes the atheronecrotic core to coagulation factors and platelets circulating into the arterial blood and induces arterial thrombosis, thus leading to an acute cardiovascular event (i.e., acute myocardial infarction, stroke).

A growing body of evidence defines atherosclerosis as a complex and systemic pathology in which hyperlipidaemia is a significant factor; the persistence of inflammation is required for plaque evolution and destabilization and plays a decisive role in the pathogenesis and worsening of coronary artery disease.

In the past two decades, research into the genetic basis of coronary heart disease has intensified, although clear evidence regarding the heritability of complex diseases such as coronary artery disease (CAD) is missing (21). Surprisingly, as observed by Libby et al. almost 75% of CHD single nucleotide polymorphisms occur in or near genes with no direct connections to atherothrombotic mechanisms, obscuring our understanding of the functions of relevant genes (22). More recently, research has focused on epigenetic variability (i.e., variations in gene expression without variations in genome structure) and the importance of activating/inhibiting different epigenetic sites related to immune-related processes involved in CAD pathogenesis (23).

## The heart as an immune organ

According to the most recent research, the heart can respond to acute tissue injury through complex inflammatory and reparative cascades, acting as an “immune organ” (24).

Several types of immune cells, such as macrophages, dendritic cells, mast cells (MCs) and a small number of B and T lymphocytes, reside in the heart. Irrespective of the origin of cell injury, acute tissue damage promotes an inflammatory response, mainly characterized by the involvement of innate immune cells, followed by a reparative/remodeling phase, with a prevalence of adaptive immune cells and angiogenic/fibrotic events (25).

### The inflammatory cascade in ischaemic myocardial injury

Histological patterns that characterize acute myocardial infarction include coagulative necrosis as the main process, regulated necrosis (necroptosis), and/or secondary apoptosis.

**Figure 2:**
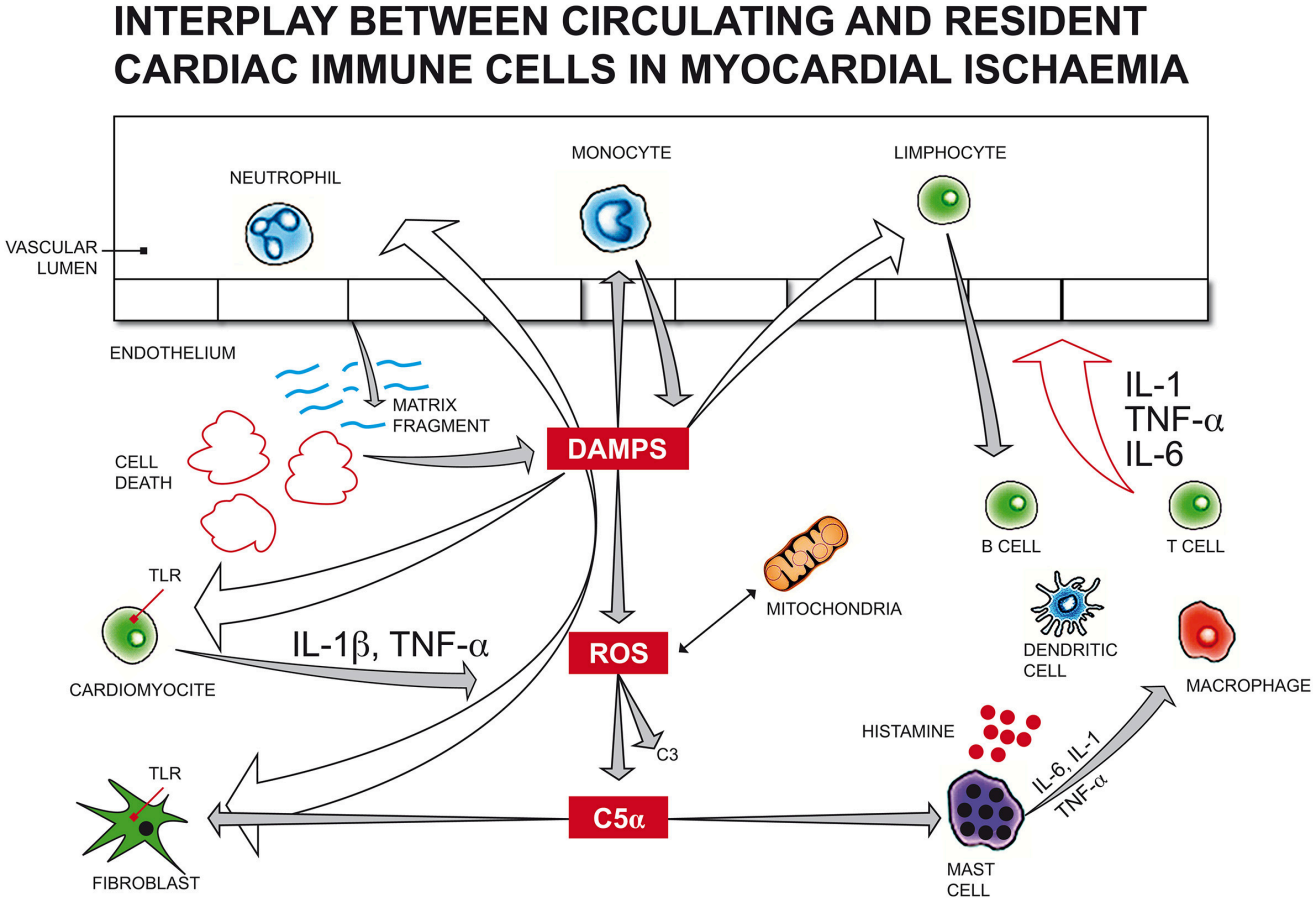
Following myocardial infarction, cellular fragments released by dying or injured myocardial cells can trigger resident cardiac immune cells as endogenous “alarmins,” similar to microbial pathogen-associated molecular patterns (PAMPs) or damage-associated molecular patterns (DAMPs), through the engagement of membrane toll-like receptors (TLRs). Endogenous DAMPs can also activate the complement system, as observed with increased C3, C5 fragments in infarcted myocardial tissue, promoting leukocyte translocation in injured myocardium. Immune activation by DAMPs can arise also from mitochondrial DNA released by the mitochondria of damaged cardiac cells due to haemodynamic stress, inducing cardiomyocyte apoptosis. The generation of a large amount of reactive oxygen species (ROS) from mitochondria impairs myocardial function and activates leukocyte migration, reducing the number of vital cardiomyocytes and promoting extracellular matrix degradation. Many mediators of different origins can activate mast cells (MC) degranulation. In the first 24 h after an acute ischaemic cardiac event, endogenous DAMPs induce the release of histamine, TNF-α, IL-6, prostaglandins, leukotrienes, tryptase, chymase and renin from MCs, leading to an acute immune response, which, in turn, actives resident immune cells, like macrophages, T, B, and dendritic cells. DAMPs, damage-associated molecular patterns; ROS, reactive oxygen species; C5α, complement component 5 alfa; C3, complement component 3; TNF, tumor necrosis factor; IL-1/IL-1β, Interleukin 1/Interleukin 1 beta; TLR, toll-like receptor; IL-6, Interleukin 6.

Following myocardial infarction (Figure 2), cellular fragments released by dying or injured myocardial cells can trigger resident cardiac immune cells as endogenous “alarmins,” similar to microbial pathogen-associated molecular patterns (PAMPs) or damage-associated molecular patterns (DAMPs), through the engagement of pattern recognition receptors (PRRs) such as membrane toll-like receptors (TLRs) and intracellular nucleotide binding and oligomerization domain (NOD)-like receptors (NLRs).

In human hearts, TLR2 and TLR4 are the most representative subtypes and, together with NOD1 and the NLRP3 subtype, have been demonstrated to activate inflammasomes in the heart (26) and play critical roles in cardiac remodeling following myocardial infarction and ischaemia/reperfusion damage (27).

Endogenous DAMPs can also activate the complement system, as observed with increased C3 fragments in infarcted myocardial tissue, promoting leukocyte translocation in injured myocardium.

Immune activation by DAMPs can arise also from mitochondrial DNA released by the mitochondria of damaged cardiac cells due to haemodynamic stress, inducing cardiomyocyte apoptosis. Additionally, ischemic damage leads to mitochondrial dysfunction, accompanied by the generation of a large amount of reactive oxygen species (ROS). Exorbitant production of ROS impairs myocardial function and activates leukocyte migration, reducing the number of vital cardiomyocytes and promoting extracellular matrix degradation (28).

Nuclear factor kB (NF-kB), a highly conserved DNA transcription factor, drives the production of cytokines, interferons, and chemokines in the myocardial infarction zone when activated via the TLR and NOD pathways, complement system, and ROS products (29).

In a vicious circle, interleukin (IL)-1 and tumor necrosis factor-alpha (TNF-α), the main pro-inflammatory cytokines released in the injured myocardium, mediate the synthesis of more chemotactic factors that enhance leukocyte recruitment into the infarct area and play important roles in inflammasome assembly and IL-1β and interleukin 18 (IL-18) maturation (30).

### Mast cells and cardiac events

Mast (or mastoid) cells (MCs) are innate immune cells located in mucosal and epithelial tissues throughout the body (including the brain and heart).

MCs, accumulating in the arterial adventitial space in response to mechanical or hypoxic stress, play a critical role in the fibrous cap erosion of atherosclerotic plaques and in coronary vasospasms induced by vasoactive and inflammatory mediators (31). Coronary vessels of patients with ischaemic heart disease have higher numbers of mast cells and histamine-rich granules than non-atherosclerotic vessels (32). Many mediators of different origins can activate MC degranulation: macrophages that engulf LDL-ox, complement fragment 5a (C5a), IL-1, and ROS.

In the first 24 h after an acute ischaemic cardiac event, endogenous DAMPs induce the release of histamine, TNF-α, IL-6, prostaglandins, leukotrienes, tryptase, chymase and renin from MCs, leading to an acute immune response (sterile inflammation). Preformed MC mediators and DAMPs from necrotic cardiomyocytes induce neutrophil recruitment, tissue digestion through protease secretion and the phagocytosis of necrotic cells and induction of apoptosis in live cardiomyocytes, thus amplifying the inflammatory response (24).

### Neutrophils in ischaemic injury

Neutrophils are the most prevalent and efficacious leukocytes of innate immunity in preventing pathogens dissemination. Both cardiac ischaemic injury and pressure overload can immediately activate neutrophil recruitment in the myocardium. Mitochondrial DAMPs of ischaemic cardiomyocytes mediate neutrophil homing via TLR patterns. Pro-inflammatory cytokines (i.e., TNF-α, IL-1β) and chemokines released by tissue- and monocyte-derived macrophages, as well as histamine released by mast cells, can stimulate the cardiac endothelium to express adhesion molecules, favoring further neutrophil transmigration into damaged cardiac tissue. IL-6 is another inflammatory mediator released by neutrophils, monocytes, and cardiomyocytes themselves and enhances leucocyte cytotoxic activity and recruitment. Notably, neutrophils play a decisive role in ischaemia/reperfusion injury through the production of proteases that significantly impair cardiac function (33).

### Monocytes, tissue macrophages, and myocardial infarction

The immune response to acute myocardial infarction includes an inflammatory phase at days 1–4 and a healing phase from days 4 to 15. In a seminal study, (34) identified two subsets of monocytes, Ly6C^Hi^ and Ly6C^Low^, that were recruited sequentially via CCR2 and CX3CR1, respectively. In the early phase, Ly6C^Hi^ monocytes removed necrotic tissue exhibiting phagocytic, proteolytic, and inflammatory properties. Ly6C^Low^ monocytes indeed promote tissue healing via myofibroblast maturation, the deposition of collagen fibers, and vascular angiogenesis through increased endothelial growth factor (VEGF) expression. Monocyte recruitment in the human heart depends on innate B cells, which drive monocyte expansion in a CCL7-dependent fashion once they are recruited into ischaemic cardiac tissue.

The pro-inflammatory subset of monocytes in humans express CD14^++^CD16^−^, and pro-healing monocytes express CD14^+^CD16^++^ (25).

Recent evidences (35, 36) have shown that resident cardiac macrophages encompass three different cell subtypes based on differential cell surface receptor expression (major histocompatibility class II, MHC-II, and C-C chemokine receptor 2, CCR2): MHC-II ^Hi^ and MHC-II ^Low^ macrophages of embryonic origin, and CCR2^+^ macrophages derived from peripheral blood monocytes. During the neonatal period, the abundance of MHC-II ^Hi^ and MHC-II ^Low^ macrophages allows the regeneration of cardiac tissue in response to tissue injury, unlike the adult period when the prevalence of CCR2^+^ macrophages permits minimal regeneration processes and/or progression toward pathologic cardiac remodeling following ischaemic damage.

MHC-II ^Hi^ and MHC-II ^Low^ macrophages phagocytize apoptotic cells, promote angiogenesis and render neonatal cardiomyocytes more responsive to proliferative stimuli through minimal inflammation and more efficient than non-embryonic macrophages, although data on adult cells are less definitive (37). In adult myocardial infarction, there are large numbers of CCR2^+^ macrophages and monocyte expansion that drives inflammatory responses through inflammasome activation and oxidative damage, thus impacting cell healing.

In experimental models, 24–96 h after a cardiac ischaemic event, Ly6C^Hi^ monocytes, together with IgM/IgD^+^ innate B cells that amplify monocyte recruitment, transmigrate into the ischaemic area. Zouggari et al. (38) Monocytes internalize apoptotic immune cells and secrete other inflammatory mediators, in particular IL-23, which drives innate γδ T cells to produce IL-17a, thus resulting in further cardiac cell death (24).

### T and B cells and cardiac events

T helper cells (Th), which are T lymphocytes expressing CD4^+^ receptor on the surface, have a fundamental role in the adaptive immune response, helping T and B lymphocytes to secrete cytokines and polarizing immune activity toward a humoural, cytotoxic or regulatory response. Th cells have traditionally been classified into Th1, Th2, Th17, and T-reg subsets based on the effector cytokines released, with a prevalence of IL-12 and IFN-γ in Th1, IL-4, and IL-13 in Th2, IL-17, and IL-23 in Th17, and finally IL-10 and TGF-β in T-regs. (39)

These subsets are involved in the pathophysiology of several chronic diseases, including heart-related diseases. In animal models, following acute myocardial infarction, an imbalance in the Th1/Th2 subpopulation toward Th1 has been observed (40). Th1-Th17 polarization has also been observed in atherosclerotic patients and in subjects hospitalized for decompensated heart failure. On the other hand, the prominence of Th2 and T-reg cells has been reported to have a protective effect both in acute myocardial infarction and heart failure (41).

T cells are also implicated in the aging heart in the absence of cardiac events. In a recent study (42), Ramos et al., showed that T cells in elderly subjects are responsible for cardiac inflammation and functional impairment, even in the absence of organ damage or ongoing infection.

B lymphocytes, both tissue-resident and circulating cells, play a fundamental role in heart homeostasis. The B2 subset has been classified as pro-atherogenic, whereas the B1 subset has an athero-protective role and drives LDL-ox removal through binding with IgM antibodies (43).

Following an ischaemic cardiac event, myocardial proteins released in the bloodstream activate dendritic cells (DC) and other antigen presenting cells (APCs) that interact with memory B cells, leading to clonal expansion and the synthesis of auto-antibodies, which can initiate the apoptosis of live cardiomyocytes. Naïve B cells can perpetuate cell damage through the release of inflammatory cytokines and chemokines that recruit monocytes, as well as T lymphocytes through MHC and T cell receptor interaction (41).

This new knowledge regarding the immunity of acute cardiovascular disease has oriented experimental therapeutic applications toward the blockage of inflammatory mediators such as TNF-α or IL-1, yielding inconclusive outcomes. Promising strategies for patients with severe heart failure and ventricular dysfunction are targeted to the selective removal or neutralization of circulating antibodies against adrenergic receptor beta-1 (anti-β1 AR), which exerts cardiotoxic effects (44).

### Summary: the balance of inflammatory responses in ischaemic cardiac disease

The presence of immune cells in an infarcted area is essential for the initiation of repair processes in injured cardiac tissue. In the first hours after ischaemic damage, the activation of resident immune cells and recruitment of leukocytes from the circulation aims to remove dead cells, molecular fragments and debris, and release cytokines and growth factors to build highly vascularized granulation tissue to maintain the integrity of the myocardial wall. The subsequent reparative phase characterizes the days and weeks after myocardial infarction, with the formation of fibrotic scars and new vessels in the injured area.

Temporal and spatial regulation of post-infarction inflammatory responses is crucial. Patients of different ages with diverse comorbidities (i.e., diabetes, hypertension) and genetic characteristics may have different remodeling responses, irrespective of the size of the myocardial necrosis. Exorbitant early inflammatory signals may enhance extracellular matrix lysis, leading to cardiac rupture. Prolonged inflammation or defects in the regulatory feedback of inflammatory mediators may reduce collagen deposition and provoke dilatation of the cardiac chambers. Poor control of the inflammatory reaction may extend the immune infiltrate beyond the non-infarcted myocardium, increasing matrix protein deposition and worsening fibrosis and diastolic function (29).

Despite improvements in clinical management and the survival rate in recent years, 12% of patients die within 6 months after acute myocardial infarction, and inflammation takes a central role in atherosclerotic plaque destabilization. Identifying a high-risk pattern of circulating inflammatory cells may have prognostic value for secondary prevention in ischaemic patients. According to Meeuwsen's recent review, elevated numbers of neutrophils appear to be consistently related to adverse outcomes in CAD and post-AMI patients and, despite the limited existing data, adds significant prognostic value in addition to common prediction calculators such as GRACE and the TIMI score (45).

## The heart as an endocrine gland

In the middle of the last century, granules similar to those found in endocrine cells were detected for the first time in the cardiac atria, and their ability to lower blood pressure through potent diuretic and natriuretic effects was proven at the end of the 1970s. Only in the early eighties (46, 47) were the granules also identified in other tissues and classified into three main subtypes: atrial natriuretic peptide (ANP), brain natriuretic peptide (BNP) and type C natriuretic peptide (CNP).

More recently, the discovery of other endocrine molecules (collectively named cardiokines) released by cardiomyocytes, fibroblasts, vascular endothelial cardiac cells and immune cells was a significant advance in research investigating the endocrine properties of the heart (48).

Any haemodynamic condition determining atrial distention (i.e., volume expansion with saline solution, water immersion, postural changes, a high quantity of ingested salt, fluid overload due to diastolic cardiac impairment) increases ANP release into the bloodstream. Chemical stimuli such as endothelin, platelet-activating factor, corticotropin-releasing hormone (CRH), and glucagon-like peptide can also trigger the synthesis of ANP.

Brain natriuretic peptide (BNP), originally described in the pig brain, is highly expressed in the ventricular myocardium and responds to ventricular wall distension due to volume or pressure overload.

C-type natriuretic peptide (CNP) is diffused throughout the central nervous system, as well as in the vascular endothelium. This peptide exerts a weak endocrine natriuretic effect and acts as a paracrine factor for the control of vascular tone (49).

Natriuretic peptides, through increased urinary sodium excretion and vasodilation, can maintain extracellular fluid homeostasis and control blood pressure boosters. In addition to this traditional function, the ability of atrial peptides to stimulate lipolysis and lipid β-oxidation and increase energy usage through ATP synthesis in cardiomyocytes was recently demonstrated.

Indeed, natriuretic peptides promote the transformation of classical white adipocytes into beige/brown adipocytes (a process called “beiging” or “browning” according to different authors), in this way increasing heat production (thermogenesis) via crosstalk with the sympathetic nervous system (50).

Brown adipose tissue (or “brown fat”) is abundant in newborns and tends to diminish in adulthood. It is detected around great vessels (the aorta and common carotid artery), the brachiocephalic artery, paracardial mediastinal fat, the epicardial coronary artery and veins, the internal mammary artery, and the intercostal artery and veins, as well as in the intrascapular area. In contrast to most representative “white fat,” brown adipose tissue is not specialized in storing energy as fat but instead tends to “burn” lipids, producing heat through alternative catabolic pathways (i.e., uncoupling reactions).

Indeed, the heart is an organ with high metabolic activity and high energy demands due to its unremitting work; under adverse climatic conditions (i.e., exposure to low temperatures), maintenance of a normal internal body temperature is crucial for heart functions. Prolonged cold exposure is associated with impaired cardiac performance related to the volume-overload hypertrophy of ventricles. Under these conditions, there is an increase in circulating natriuretic peptides with a parallel enhancement in fatty acid mobilization and catabolism, higher mitochondrial metabolic activation in the cardiac skeletal muscles, and increased heat production to maintain an optimal body temperature (50).

### Therapeutic applications in progress

In an experimental context, the controlled infusion of ANP, BNP, and CNP leads to cardiovascular effects, inducing vasodilatation and lowering blood pressure. The administration of ANP, BNP, or CNP may also limit post-infarction ventricular remodeling. In decompensated heart failure, ANP and BNP infusion reduces both pulmonary capillary wedge pressure and systemic vascular resistance, thus enhancing stroke volume [for review: (51, 52)].

Natriuretic peptides exert beneficial effects on vascular resistance and fluid balance through inhibition of the renin-angiotensin-aldosterone (RAAS) axis, known to be deeply involved in cardiac remodeling in hypertensive and post-ischaemic patients. Local renin-angiotensin production in different tissues, including the heart, vasculature and brain, was recently recognized (53). Stress, through sympathetic branch activation, enhances both central and local RAAS; if chronic, stress may induce haemodynamic and pro-atherogenic modifications.

Therapeutic strategies targeting atrial peptide modulation and RAAS downregulation may include lifestyle modifications (i.e., stress management: see below) and drugs.

Classical drugs recommended for the management of heart failure include angiotensin-converting-enzyme (ACE) inhibitors, angiotensin II-receptor blockers (ARBs), β-blockers, digoxin, diuretics, and aldosterone antagonists. Sacubitril/valsartan (Entresto®;) is the first-in-class angiotensin receptor neprilysin inhibitor (ARNI) approved by the FDA in 2015 and is recommended by the latest European and American heart failure treatment guidelines (54) for patients with heart failure. Sacubitril/valsartan suppresses RAAS activation by blocking angiotensin II type 1 receptors and enhances natriuretic peptide availability through inhibition of the degrading enzyme neprilysin. Because of its recent commercialization, caution is still requested given the risk of long-term side effects on cognitive function. In fact, neprilysin is a principal peptidase involved in the degradation of β-amyloid (Aβ) protein, and further research is needed to determine the risk of developing Alzheimer's disease in patients with mild cognitive impairment (55).

## Heart and stress system

The natural history of CHD can be influenced by mental stress. First observations of negative emotions and coronary thrombosis were provided in 1910 by Obraztsov and Strazhesko, who included for the first time emotional triggers in the pathogenesis of acute myocardial infarction (56).

Stressful life events, such as natural calamities, financial crises, terroristic attacks and wars, are known to be life-threatening acute triggers for cardiac events, as are positive emotionally charged events (sport matches and Christmas and New Year's holidays), thus worsening the prognosis in vulnerable individuals (57).

### Psychosocial factors as independent risk factors for cardiac diseases

Chronic stressors such as negative psychosocial factors represent modifiable risk factors that could be linked to adverse cardiac prognosis and the mortality rate worldwide. The international INTERHEART case control study proved that psychosocial factors were significantly related to acute myocardial infarction, with an odds ratio (OD) of 2.67 (58).

Social inequalities and behavioral factors as determinants of CV morbidity and mortality were also investigated by M. Marmot and colleagues (59) in a cohort of British civil servants who worked in the late 1960s (the Whitehall I study) and in 1985–88 (the Whitehall II study). The results from these long-term prospective studies, initially considered platforms for studying age-related diseases, for the first time linked lower socioeconomic status (SES) and lower employment grade with a higher incidence of metabolic syndrome stigmata and a higher coronary mortality rate among male employers. Other combined variables associated with increased risk of CVD mortality were high-strain work (low control and high demands) and low social support. In the same cohort, the presence of financial difficulties in lower employment grades was a risk factor for weight gain and metabolic alterations, in particular in female workers. Findings derived from these large cohort studies clearly show the direct correlation between social conditions and metabolic disturbances, coronary disease onset and the mortality rate (60).

Further meta-analyses of prospective observational studies found that certain psychosocial factors, such as social isolation and loneliness, were associated with a 50% increased risk of CVD; work-related stress showed similar results, with a 40% risk of new CV events (61).

Experimental data confirmed that adverse early life events, including social deprivation and discrimination during childhood and adolescence, predispose an individual toward the development of CVD in adulthood through diverse epigenetic signatures of key regulatory genes involved in the stress response, immune function, inflammation and metabolism (62).

However, the lack of statistical power in recent metanalyses does not allow identification of the type of occupational psychosocial factors that can be considered independent risk factors for major cardiac events (63).

### Emotions and cardiovascular disease

As emerged by large observational studies, people with severe mental diseases (i.e., schizophrenia, bipolar disorder, and major depressive disorder) have an increased risk of developing CHD compared with controls, with pooled hazard ratio of 1.54, according to recent meta-analytic results (64), showing a consistent increase in cardiovascular morbidity and mortality.

In 2014, the American Heart Association pointed out the close relationship between high depressive symptoms and poor prognosis after acute myocardial infarction; in a published scientific statement, depression was elevated as an “independent primary risk factor in patients with acute coronary syndrome” (65, 66). In fact, the incidence of coronary heart disease was measured at a relative risk of 1.90 in the presence of diagnosed depression (67, 68).

Racial disparities have been considered in many prospective studies, recognizing race-dependent risk factors for blacks, but not whites, in developing cardiac disease. In the REGARDS study, black individuals with depressive symptoms presented a greater risk of CHD diagnosis or revascularization at follow-up (69). In the 10 years of follow-up in the Jackson Heart prospective study, the presence of depressive symptoms was positively correlated with the risk of incident stroke. However, coping strategies observed in some individuals of the cohort may mitigate the increased CHD risk associated with depressive symptoms (70).

Anxiety is commonly diagnosed together with depressive disorder. Therefore, it is not surprising that there are few studies focusing only on anxiety disturbance and the incidence of cardiovascular disease. In a 2010 meta-analysis by Roest and colleagues, a high anxiety score was a recognized risk factor linked to coronaropathy, although the analysis was not adjusted for depression, a common comorbid disease (71). In a cohort of thousands of young Swedish military men, those who were diagnosed with anxiety were more likely to experience coronary heart disease and myocardial infarction (72). Seven years of follow-up in Finnish longitudinal study conducted on healthy people reported an association between anxiety and elevated risk of CHD in women only (73).

The link between posttraumatic stress disorder (PTSD) and incident fatal and non-fatal CVD events is well established. Diagnosis of PTSD was found to be an established risk factor for acute coronary events in the general population in multiple prospective cohort studies (74) and in subgroup population studies involving veterans (75) and women (76).

Positive thoughts and emotions, as well as social cohesion, enhance resilience and influence health trajectories in cardiovascular diseases. In the Health and Retirement Study, optimism appeared to protect against incident heart failure after a cardiac event (77).

Ethnic differences in positive behavioral responses emerged in the Eastern Collaborative Group prospective study, in which the Japanese attitude called “Spirit of Wa,” integrating a sense of community, collaboration and hierarchical social organization, was a protective factor against further cardiac events in Japanese men undergoing coronary angiogram for CAD (78). Assessment of baseline coping strategies in another cohort of hypertensive middle-aged Japanese subjects without a history of CVD demonstrated that individuals who presented an approach-oriented coping strategy were more likely to have reduced incidence of stroke and CVD mortality, while an avoidance-oriented behavior was associated with higher CVD incidence and mortality (79).

In western countries, evidence has shown similar results. Adults in the United States without cardiovascular disease who perceived higher neighbourhood social cohesion presented a reduced likelihood of incident myocardial infarction over 4 years (80).

### Stress and neuroendocrine patterns in cardiovascular disease

Stress in mammals is responsible for complex psycho-neuro-immuno-endocrine responses that primarily involves both the hypothalamic–pituitary–adrenal (HPA) axis and the autonomic nervous system (ANS), first described by the two founders of stress science, Walter Bradford Cannon and Hans Selye, in the 1930s (81, 82).

Highly conserved in all vertebrates, including humans, the ANS and HPA systems represent the neuronal and hormonal limbs of the stress response, respectively, and provide both short- and long-term changes in behavior, cardiovascular functions, endocrine and metabolic signals, as well as in host defense and immune responses, enabling the individual classically “to fight or flee” and initiate different coping strategies against stressors of different origins, from physical injuries to psychosocial tasks, in order to successfully adaptation (allostasis). The HPA axis, starting from the hypothalamic paraventricular nucleus, secretes corticotropin-releasing factor (CRF) that regulates the release of adrenocorticotropic hormone (ACTH) from the pituitary gland. Cortisol is the main active hormone released from the adrenal gland in response to ACTH in humans, exerting negative feedback control on hypothalamic CRF and pituitary ACTH secretion (83).

As observed in clinical studies with adults and adolescents, altered HPA axis function may have negative effects on the cardiovascular system, leading to atherosclerotic plaque formation, high blood pressure, insulin resistance, dyslipidaemia, and central adiposity. Biomolecular studies confirm that these stigmata correlate with elevated inflammatory markers and endothelial activation with a hypercoagulable state and increased risk of thrombotic events (84).

A growing body of evidence has demonstrated a close relationship between high levels of cortisol and increased risk of ischaemic heart disease and cardiovascular mortality (85, 86).

Chronic psychological stress is associated with the pathogenesis of atherosclerosis, and serum cortisol might be a reference marker for this disease. Huo et al., showed that serum cortisol levels were higher in the patients with atherosclerosis than in healthy controls, and high plasma cortisol concentrations negatively correlated with circulating immuno-regulatory IL-10, promoting plaque destabilization (87). Chronic job-related stress leads to metabolic syndrome. Workers suffering from burnout showed dysregulation of the sympathetic vagal balance, with reduced parasympathetic activity, predominance of sympathetic activity, and hyporeactivity of the HPA axis, mainly in males (88).

Results from the Whitehall II study showed that male workers with metabolic syndrome at lower job positions had higher levels of norepinephrine, cortisol and serum IL-6 and manifested a higher heart rate at rest and lower heart rate variability (89).

As a systemic disease, obesity itself contributes to the risk for CVD through elevations in basal levels of cortisol, inflammatory cytokines and hormones such as leptin and insulin. In an exiguous group of obese college-aged males, Caslin and colleagues showed that an acute mental stress task elicited a vigorous stress reaction, with an increase in heart rate and catecholamine release (epinephrine and norepinephrine), increased immune response with inflammatory cytokine synthesis (TNF-α, IL-1 and IL-6) and hormonal changes with a significant reduction in leptin concentrations, without a significant increase in serum cortisol at an early post-task observation time point (90).

### Circadian rhythms and cardiovascular health

Life has evolved on earth under the influence of the alternation of the day-night cycle. Humans, such as other primates, synchronize on this rhythm the variable (circadian) production of hormones, neurotransmitters, and cytokines, as well as brain area activation and the main functions of fundamental organs, such as the pancreas, liver, adrenal glands, bowel, lungs and heart (91).

Sleep is the most important circadian synchronizer in human organisms, and sleep deprivation, due to lifestyle habits or related to organic conditions such as obstructive apnoea, insomnia or neurologic and psychiatric diseases, affects modern societies worldwide. Although most pathophysiological mechanisms are still poorly understood, the biological effects of alterations in the quantity and quality of nocturnal sleep have been deeply studied. Epidemiological observations have demonstrated that short sleep duration is related to increased incidence of hypertension, coronary artery disease, arrhythmias, obesity and diabetes (92).

Recent reports have documented the relationship between sleep duration and sleep quality (93) with incidence of coronary heart disease. Two large cohort studies have shown that the reduction of nocturnal sleep to 6–8 h per night significantly increases the risk of coronary artery disease, and sleeping < 4 h per night or more than 8 h per night can increase coronary events (93, 94).

The mechanisms underlying the detrimental effects of sleep disorders on cardiovascular disease are limited. In animal models, sleep deprivation alters cardiovascular parameters and increases sympathetic activity and neuroendocrine responses to experimental stressors, as well as induces inflammatory, and pro-oxidant patterns in many tissues (brain, fat, liver, spleen), including the heart and vascular endothelium (95). In humans, sleep deprivation is characterized by a more highly activated HPA axis and elevated cortisol secretion in response to an acute experimental stress task (i.e., Trier Social Stress Test) (96). Subjects included in the Multi-Ethnic Study of Atherosclerosis who slept < 6 h per night showed impaired cardiac autonomic regulation (measured through the heart rate variability index, HRV), with higher levels of sympathetic tone and lower levels of parasympathetic tone compared to those who slept 7 h or more per night (97).

One single sleepless night can affect the cardiovascular autonomic response toward sympathetic predominance and induce systemic inflammation through increased IFN-γ secretion in young internists after one night of on-call duty (98). Recent findings reported an increase in circulating IL-6 and von Willebrand factor (pro-thrombotic factor) related to lower sleep efficiency among women aged 40–60 (99).

### Autonomic regulation of cardiac function: the neuro-cardiac axis

The autonomic nervous system (ANS) maintains homeostatic equilibrium in response to normal physiological stimuli such as environmental changes (standing, exercise, and temperature modification) or to pathological conditions such as physical diseases and mental stress. The link between stress and CVD occurrence is mediated by autonomic dysregulation through central and peripheral neurophysiologic mechanisms involved in the onset of arrhythmic and acute coronary events, as well as worsening heart failure.

The neuro-cardiac axis is a complex series of reflex control networks containing afferent, efferent, and local circuit neurons that coordinate, in an interdependent fashion, cardiac electrical and mechanical properties (100) by mediating autonomic responses to stressful stimuli.

The central cardiac control network involves cortical centers, mainly the insular cortex (IC), anterior cingulate cortex (ACC), amygdala (A), hypothalamic nuclei, medulla, and spinal cord. The medulla, located in the lower segment of the brainstem, contains sympathetic and parasympathetic nuclei that directly regulate autonomic nerve efferences to the heart and blood vessels. The nucleus tractus solitarius (NTS) of the medulla receives afferences both from peripheral baroreceptors and chemoreceptors and from the hypothalamus and other cortical regions, directly modulating the activity of vagal and sympathetic innervations. From the medulla, the sympathetic fibers come down the spinal cord, where they immediately make synapses with preganglionic fibers. The sympathetic preganglionic efferent fibers arise in the intermediolateral column (IML) of the spinal cord, receive excitatory glutamatergic inputs in rostral ventrolateral medulla (RVLM) and make synapses in several extracardiac neuronal ganglia (i.e., stellate and middle cervical ganglia). Sympathetic postganglionic efferent fibers finally form synapses in the heart and vasculature.

The parasympathetic nerves, primarily located in the nucleus ambiguus (NAmb) and in the dorsal motor nucleus of the vagus (DMN), exit the medulla as long preganglionic efferent fibers forming the vagus nerve or cranial nerve X and finally synapse with postganglionic fibers within the heart or vascular tissue. Vagal and adrenergic postganglionic fibers, together with local circuit neurons, represent the intrinsic cardiac nervous (ICN) system, a complex network organized in clusters of cardiac plexuses that influence the cardiac conductile tissue to affect the heart rate, myocardial contractions and atrioventricular conduction velocity (101).

It is well known that acute injury occurring in the central nervous system (both in cortical regions and in the brainstem) is linked to transient or permanent myocardial and/or arrhythmic damage. Ischaemic damage to the insular cortex, in particular right-sided lesions, is associated with arrhythmic alterations (atrial fibrillation, atrioventricular block, ectopic beats, sinus bradycardia), inverted T wave, sudden cardiac death, altered diurnal blood pressure, and myocardial injury, as well as elevated plasma levels of brain natriuretic peptide, catecholamines, and sympathetic neuropeptide Y (NPY) (102). NPY is released as ancillary neuropeptide from the sympathetic nerves of the heart during very high rates of nerve firing. NPY release is also demonstrable in patients with panic disorder during an acute attack. NPY is highly vasoconstrictive, causing coronary artery spasms, and might be eventually related to the development of stress-related cardiomyopathy (103).

Stress exposure, through its deleterious impact on the autonomic cardiac system, leads to an imbalance in autonomic regulation (i.e., sustained sympathetic activity and/or reactivity, delayed sympathetic recovery, reduced vagal activity), resulting in a dramatic shift toward enhanced sympathetic firing and/or withdrawal of vagal tone and higher risks of hypo- and hyperkinetic arrhythmias, platelet aggregation, and vascular thrombosis. It has been experimentally observed that sympathetic stimulation triggers cardiac arrhythmias, reducing both atrial and ventricular arrhythmic thresholds, and induces ECG changes in the repolarization phase (104). In patients with implantable defibrillators, mental stress can induce T wave changes at lower heart rates than that achieved with physical exercise, as observed by Kop and collaborators (105).

The stellate ganglia also play an important role in the generation of ventricular and atrial arrhythmias. During experimental sympathetic discharge, stellate ganglia activation induces T wave changes. Removal of the caudal portions of both stellate ganglia suppresses ventricular arrhythmia formation in humans (106).

Esler and co-workers have demonstrated a close relationship between stress exposure and essential hypertension. In an experimental setting, patients affected by panic disorder without an acute panic attack showed multiple “salvoes” of sympathetic nerve firing during normal cardiac activity; researchers found an identical pattern in hypertensive patients, identifying sympathetic firing as a “signature” of chronic stress exposure (107). The co-release of adrenaline and noradrenaline from the sympathetic fiber in the hearts was also observed in both hypertensive patients and individuals with panic attacks, as well as in experimental models of mental stress, but not in healthy people. Isotope and immunoblot staining methods have demonstrated that both in essential hypertension and in panic disorder, the adrenaline synthesizing enzyme phenylethanolamine N-methyltransferase (PNMT) synthetizes approximately 10% of the total amount of catecholamine released by the cardiac sympathetic nerves (108).

In patients suffering from heart failure, autonomic dysregulation correlates with higher plasma NE concentrations and might predict negative outcomes (109).

Heart rate variability (HRV) represents a non-invasive marker of life-threatening arrhythmias and vascular endothelial damage, as well as an indirect marker of chronic stress and emotional arousal. HRV indicates a dynamic variation in the time interval between heartbeats, used as an index of sympathetic activity that affects the variability of the cardiac rate, in general, reduced HRV indicates excessive sympathetic activity and depressed vagal tone. Psycho-social stress tasks may result in chronic sympathetic activation, thus influencing HRV. It has been observed in different cardiac and non-cardiac diseases that reduced HRV negatively affects health outcomes. In the presence of adverse psychosocial factors, there is an autonomic system imbalance with reduced HRV and increased carotid stiffness in post-menopausal women (110). According to reviews by Guan and collaborators, ANS dysfunction and impaired HRV may represent independent risk factors for subsequent ischemic stroke after TIA or minor stroke (111).

### The inflammatory reflex of the vagus nerve

Experimental distress is found to be linked not only to autonomic nervous system imbalance but also to enhanced inflammatory activity by myocardial macrophages, resulting in cardiac dysfunction and cardiomyocyte death. In 2000, Tracey and colleagues discovered “the inflammatory reflex,” a characteristic of vagal activity in counteracting sympathetic hyperactivation; these researchers demonstrated that vagus nerve stimulation was able to immediately suppress cytokine synthesis in an animal model of sepsis. Immunofluorescence assays and biomolecular findings have shown that vagal efferent innervation mediates acetylcholine (ACh) release both in the reticulo-endothelial system and in the heart, where the neurotransmitter binds the nicotinic receptor (nAChr) expressed on resident cardiac macrophages, blocking the release of the main pro-inflammatory cytokines (i.e., TNF-α, IL-1) (112). Non-invasive vagus stimulation can be elicited through mind-body techniques (i.e., yoga, physical relaxation, meditation, biofeedback) (113).

### Quantification of myocardial stress: mental stress-induced myocardial ischaemia

Mental Stress-Induced Myocardial Ischaemia (MSIMI) is a corollary of different validated techniques used to assess the effects of stress on cardiac function (114).

In contrast to exercise and pharmacologic stress-induced ischaemia, MSIMI is a provocative test that provides psychological rather than physical stimuli, leading to reversible myocardial ischaemic damage. Different mental stressors (mnemonic, cognitive, emotional strains) are used to recreate behavioral challenges experienced in everyday life (115).

Many different instrumental resources have been tested: ECG recording, echocardiography, ventriculography, myocardial scintigraphy, positron-emission tomography, and coronary angiography (116).

The following heterogenic haemodynamic features associated with MSIMI result in myocardial transient ischaemia: increased resistance of vascular walls, coronary vasospasm, reduced endothelial function, elevated heart rate and/or blood pressure, anomalies in the electrical repolarization phase, ventricular kinetics and myocardial perfusion. Moreover, mental stress-induced activation of the stress-response system increases catecholamine release, leading to cardiac electrical instability (117). Subjects with an exaggerated cardiovascular response during mental stress were also more likely to present pathologic findings in a classic exercise/pharmacologic stress test (118).

Conversely, the presence of depressive symptoms in young post-myocardial infarction patients was associated with a higher tendency to manifest ischaemic features with mental stress rather than with exercise or a pharmacological stress test (119).

As confirmed in recent metanalyses, a positive MSIMI test represented a negative prognostic factor for adverse outcomes in CAD patients, irrespective of the coronary grade of stenosis. (117).

### Myocardial infarction with normal coronary arteries: takotsubo stress cardiomyopathy

As shown in recent epidemiological data, the diagnosis of myocardial infarction with normal coronary arteries (MINCA) has increased, constituting 5–25% of total myocardial infarction diagnoses. Approximately 30% of total MINCA is represented by Takotsubo stress cardiomyopathy (TTC), an acute systolic and diastolic dysfunction of the left ventricle that occurs in patients without a history of CAD, although a small fraction of patients may have asymptomatic CAD subsequently discovered with angiography. TTC predominantly affects elderly women (with a prevalence of men in Japan, where TTC was first described) and is often preceded by an acute emotional trigger (i.e., bereavement, separation), albeit physical triggers (i.e., accidents, earthquakes) or sine causa TTC may also occur. The etiology of TTC is unknown; however, physiological mechanisms underlying the neuro-cardiac axis may provide a fundamental link between acute stress and TTC onset. It has been hypothesized that TTC patients experience excessive catecholamine and NPY release, with subsequent coronary vasospasm, leading to a pathological reduction in myocardial blood flow and alterations to parietal kinetics. Despite previous theorizations that defined TTC as a transient benign disease, data from the International Takotsubo Registry revealed high rates of complications, such as cardiogenic shock and death, during hospitalization, similar to incidence rates of negative outcomes in acute coronary syndrome. Ten years follow-up analysis among TTC patients showed an annual 5.6% incidence of death from any cause and a 9.9% incidence of major adverse cardiac and cerebrovascular events. Moreover, the analysis showed that young patients affected by physically triggered TTC who were comorbid with acute neurological or psychiatric diseases presented a higher risk of complications and death than elderly patients with emotional triggers (120).

## Conclusions

The current view of coronary heart disease has deeply changed (see Graphical abstract); atherosclerosis is no longer considered a simple lipid storage disorder but a systemic inflammatory disease. Inflammation is a physiological response to physical, mutagenic, infective or psychologic injury; an altered or prolonged inflammatory response may inflict serious damage upon the host. Cytokines are the fundamental mediators involved in the immune response; the cytokines IL-1, IL- 6, and TNF-α enhance inflammation, whereas IL-10 acts as an anti-inflammatory cytokine that downregulates inflammatory pathways. CRP can be used as a serologic marker of vascular inflammation and seems to be a more sensible predictive factor than serum total and LDL cholesterol for incident cardiovascular events in healthy individuals.

**Graphical Abstract:**
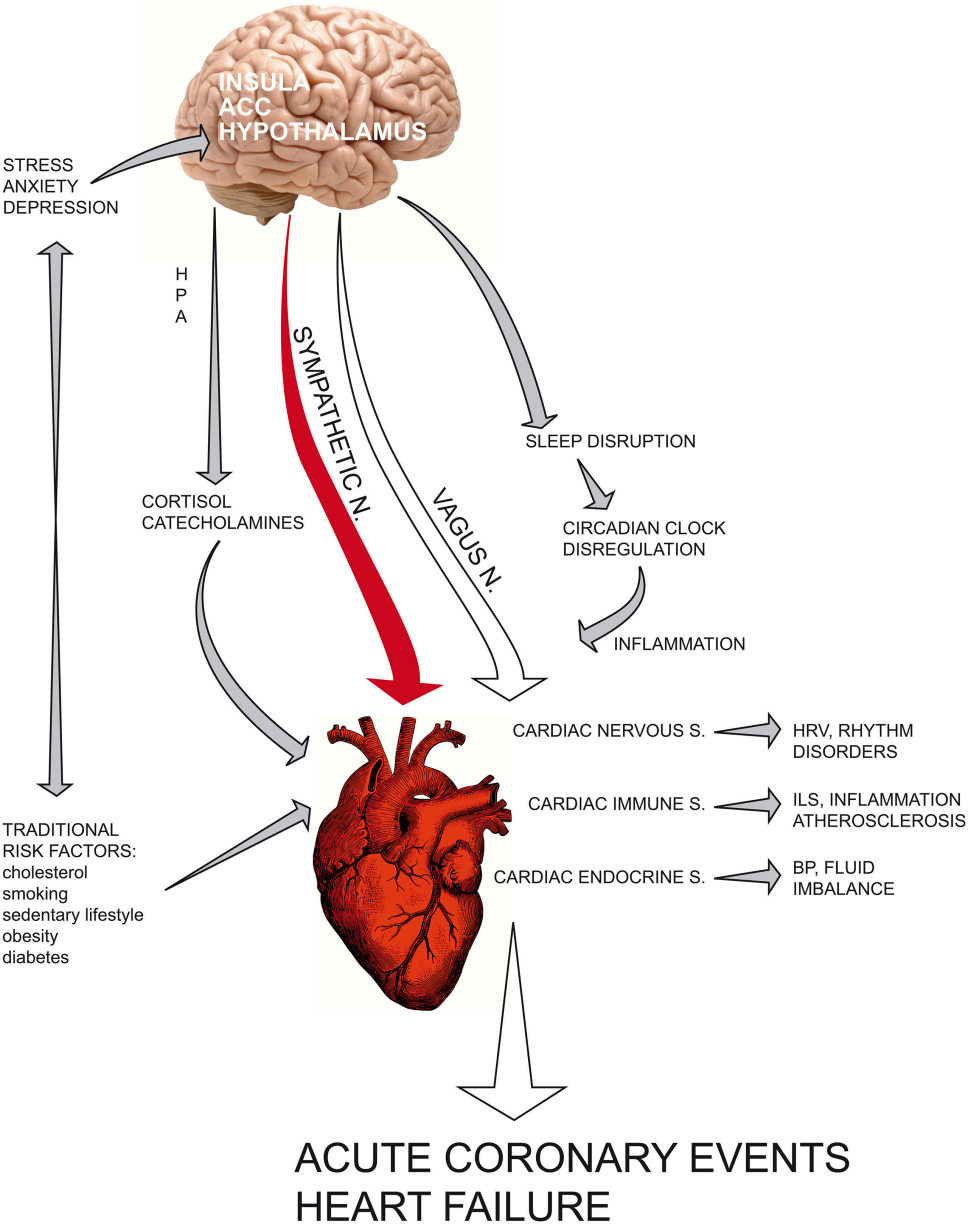
The current view of coronary heart disease has deeply changed: atherosclerosis is no longer considered a simple lipid storage disorder but a systemic inflammatory disease. Recent findings have fundamentally altered the classical vision of the heart as a “mechanical blood pump” that beats through an internal pacemaker. Indeed, cardiac tissue contains resident immune cells and is able to synthesize and release cytokines and hormones after acute myocardial infarction in the ischaemic area, influencing the healing phase. In recent years, chronic depression has ranked among the most important cardiovascular risk factors for poor prognosis in patients with myocardial infarction. Current understanding of the central and autonomic regulation of cardiac functions, namely, the neuro-cardiac axis, provides a physiological explanation that links psycho-emotional stressors and social adversities to acute cardiac event. Psychological distress can precipitate heart function through a dysregulated neuroendocrine and autonomic response. The complex network that links the heart, brain and the main biological systems provides a new vision of cardiovascular science based on psychoneuroendocrineimmunology, a science that studies the reciprocal interconnections between the psyche and nervous, immune and endocrine systems, integrating knowledge derived from the psychological and biological sciences of stress with molecular biology and epigenetic research. ACC, anterior cingulate cortex; HPA, hypothalamic–pituitary–adrenal axis; HRV, heart rate variability; ILs, interleukins; BP, blood pressure.

Recent findings have fundamentally altered the classical vision of the heart as a “mechanical blood pump” that beats through an internal pacemaker. Indeed, cardiac tissue contains resident immune cells and is able to synthesize and release cytokines and hormones after acute myocardial infarction in the ischaemic area, influencing the healing phase both in terms of acute ischaemic damage and in post-infarct myocardial remodeling.

Low-grade chronic inflammation (LGCI) is characterized by the persistence of an altered immune response with a shift toward a pro-inflammatory state, characterized by increased plasma levels of Th1/Th 17 cytokines such as interleukins IL-6, IL-1β, IL-17, and TNF-α. The increasing concentrations of these mediators result in the progressive impairment of organ functions and higher incidence of inflammation-related diseases. The pivotal role of chronic inflammation in several chronic diseases, such as asthma, cancer, diabetes, autoimmunity and psychiatric diseases including depression, is now recognized.

In recent years, chronic depression has ranked among the most important cardiovascular risk factors for poor prognosis in patients with myocardial infarction.

Current understanding of the central and autonomic regulation of cardiac functions, namely, the neuro-cardiac axis, provides a physiological explanation that links psycho-emotional stressors and social adversities to acute cardiac events, including myocardial infarction, myocardial ischaemia, cardiac wall motion abnormalities, and sudden death. Psychological distress can precipitate heart function through a dysregulated neuroendocrine and autonomic response, as demonstrated by increased serum cortisol and catecholamines, reduced HRV and increased inflammatory cascades. Sympathetic hyperactivation can influence cytokine production via the transcription of NFkB-mediated genes involved in pro-inflammatory immune response, thus enhancing vascular endothelial activation and thrombosis.

The complex network that links the heart, brain and the main biological systems provides a new vision of cardiovascular science based on psychoneuroendocrineimmunology (121), a science that studies the reciprocal interconnections between the psyche and nervous, immune and endocrine systems, integrating knowledge derived from the psychological and biological sciences of stress with molecular biology and epigenetic research (121, 122). In the next few years, the psychoneuroendocrineimmunology paradigm could make a fundamental contribution to both areas of pathogenetic research and therapeutic approaches to treat major cardiovascular diseases (123).

## Author contributions

MF, AB and FB have equally contributed to the writing of the manuscript. MB, MR, and MGR made intellectual contribution to the work. All authors approved it for publication.

### Conflict of interest statement

The authors declare that the research was conducted in the absence of any commercial or financial relationships that could be construed as a potential conflict of interest.
